# A Novel Peptide Delivers Plasmids across Blood-Brain Barrier into Neuronal Cells as a Single-Component Transfer Vector

**DOI:** 10.1371/journal.pone.0059642

**Published:** 2013-03-29

**Authors:** Ailing Fu, Miaomiao Zhang, Feiyan Gao, Xingran Xu, Zhangbao Chen

**Affiliations:** School of Pharmaceutical Sciences, Southwest University, Chongqing, China; Penn State Hershey Medical Center, United States of America

## Abstract

There is no data up to now to show that peptide can deliver plasmid into brain as a single-component transfer vector. Here we show that a novel peptide, RDP (consisted of 39 amino acids), can be exploited as an efficient plasmid vector for brain-targeting delivery. The plasmids containing Lac Z reporter gene (pVAX-Lac Z) and BDNF gene (pVAX-BDNF) are complexed with RDP and intravenously injected into mice. The results of gel retardation assay show that RDP enables to bind DNA in a dose-dependent manner, and the X-Gal staining identity that Lac Z is specifically expressed in the brain. Also, the results of Western blot and immunofluorescence staining of BDNF indicate that pVAX-BDNF complexed with RDP can be delivered into brain, and show neuroprotective properties in experimental Parkinson’s disease (PD) model. The results demonstrate that RDP enables to bind and deliver DNA into the brain, resulting in specific gene expression in the neuronal cells. This strategy provides a novel, simple and effective approach for non-viral gene therapy of brain diseases.

## Introduction

The brain-targeting gene therapy holds great promise in curing central nervous system diseases like Parkinson’s disease (PD), Alzheimer’s disease (AD), stroke and brain tumors in clinical applications [1], [2]. However, blood-brain barrier (BBB) prevents the transport of vast majority molecules from the vascular into the brain parenchyma because of its tight junctions between endothelial cells of brain capillaries [3], [4]. To overcome this barrier, the conventional approach in gene delivery is to microinjection DNA directly into the brain by stereotactic surgery, whereas this method results only in local diffusion around the injection site, also it’s invasive and unadapted for human therapy [5], [6]. Some viral vectors have been proved to be efficient in gene delivery [7], [8], but their severe side effects result in some researchers’ interest shifting to the non-viral delivery system [9]–[11].

The use of peptides as gene delivery systems is a safe alternative for gene therapy due to their advantages of biodegradability, biocompatibility, low toxicity and ease of synthesis [12], [13]. It has been reported that several arginine-rich peptides, such as (RXRRBR)_2_XB and ppTG, have the ability of mediating the oligonucleotide, siRNA or DNA delivery in vivo as non-viral vector [14]–[16]. However, there is no data up to now to show that peptide delivers plasmid across BBB into neuronal cells as a single-component gene transfer vector.

It’s well known that rabies virus glycoprotein (RVG) is the only protein component of the viral envelope that mediates viral entry into host cells [17]. The sequence analysis reveals that the 189–214 and 330–357 amino acid sequences of RVG are the important nerve binding region of the rabies virus [18], [19]. Several studies reported that RVG29-d9R (YTIWMPENPRPGTPCDIF TNSRGKRASNGGGG(d)RRRRRRRRR, derived from the 189–214 amino acid sequences of RVG), can be used to transport siRNA or cationic-polymers to the central nervous system [20], [21], but the ability of this peptide as protein or DNA transfer vector has not been tested in vivo. In our *recent* study, we demonstrated that a novel peptide, named RDP (39 amino acid residues, KSVRTWNEIIPSKGCLRVGGRCHPHVNGGGRRRRRRRRR) and derived from the 330–357 amino acid sequences of RVG, proved to have the ability of targeted delivery proteins with different molecular weight and *p*I into the brain in vivo [22]. Thus, we further assume that the RDP might have the ability to transfer the DNA into brain as non-viral vector.

Parkinson’s disease (PD) is one of the most common neurodegenerative disorders, pathologically characterized by a progressive degeneration of dopaminergic neurons in the Substantia Nigra pars compacta and their projections to the striatum. Brain-derived neurotrophic factor (BDNF) is a small dimeric protein that is widely expressed in adult mammalian brain and has been shown to promote the survival of all major neuronal types affected in the central nervous system disorders (PD, Alzheimer’s disease, depression, epilepsy and chronic pain) [23]. Studies also suggest that BDNF is a potent neuroprotective agent for therapeutic treatment of PD by rescuing dopaminergic neurons of brain [24], [25]. However, BDNF gene must be administered by intra-cerebral injection or by viral vectors, because it does not cross the BBB.

In this study, we investigate the possibility of using RDP as a single-component, non-viral transfer vector to mediate plasmids containing reporter gene Lac Z or BDNF gene into the brain. The study will provide a simple and effective approach for targeted delivery of exogenous DNA into the brain for treatment of experimental PD through systemic administration.

## Materials and Methods

### Ethics Statement

Animal experiments were carried out in strict accordance with the Chinese Guides for the Care and Use of Laboratory Animals. The protocol was approved by the Laboratory Animal Monitoring Center of Chongqing [SYXK (Yu) 2009-0002]. All efforts are made to minimize the number of animals used and their suffering.

### Plasmids

We used the plasmid pVAX-Lac Z (Invitrogen, USA) including a β-galactosidase (β-Gal) expression cassette, a CMV promoter and BGH poly(A) signal. This plasmid was purified using Promega plasmid purification kit and was quantitated by UV absorbance at 260 nm.

Plasmid pBDNF bearing human BDNF cDNA (770 bp) was purchased from Bijing Dingguo Biotech Co. Ltd (China). Primer pairs were listed as follows: P1, 5′ CAAAGCTTCAATGCACTCTGACCCTGCCCG 3′; P2, 5′ GACTCGAGCTATCTTCCCCTTTTAATGG 3′. The pfu Taq DNA polymerase was used for amplification of the BDNF gene fragments. The PCR products and plasmid pVAX-Lac Z were digested with Xho I and Hind III, and linked by T4 ligase. Then the recombinant plasmid (pVAX-BDNF) was introduced into Escherichia coli strain TOP10. The plasmids were prepared and purified using respective kits from Promega.

### Preparation of Peptide/Plasmid Complexes

For gel retardation assays, 2 µg of plasmid was incubated with RDP (synthesized by Chinapeptides Co. Ltd.,purity >99%) at 4∶1, 2∶1, 1∶1, 1∶2, 1∶4, 1∶8 gram ratios for plasmid pVAX-Lac Z (6.1 kb) to the peptide, and at 16∶1, 8∶1, 4∶1, 2∶1, 1∶1, 1∶2 gram ratios for pVAX-BDNF (3.7 kb) to the peptide, for 30 min in room temperature, subjected to electrophoresis on 1.0% agarose gels and stained with ethidium bromide. The gels were photographed using *Gel* Doc 2000 documentation system (Bio-Rad, USA). The Zeta potentials of different DNA/plasmid complexes (DNA concentration of 10 µg/ml) were measured using Zetasizer (Malvern, UK). Results are expressed as the average of three measurements.

### Serum Stability of Peptide/DNA Complex

The serum stability of the complex of DNA and plasmid was determined according to previous report [26]. Briefly, the complex (5 µg/100 µl) in 0.01 M PBS (pH 7.4) were combined with 100 µl of freshly prepared mouse serum and allowed to incubate at 37°C, then rapidly frozen at different time points. Prior to electrophoresis, SDS (10 mg/ml) was added to each aliquot along with EDTA (68 mM) and loading buffer. All samples were subjected to electrophoresis on agarose gels and detected by staining with ethidium bromide.

### Circular Dichroism (CD) Spectroscopy

CD spectra were recorded in the absence or presence of plasmids at 25°C on a Jasco J-810 spectropolarimeter (Jasco, USA), at a peptide concentration of 0.2 mg/ml in distilled water. The spectra were taken in 1-mm cuvettes at a wavelength range between 190 nm and 250 nm. All the spectra were the cumulative average of 4 repeated scans..

### Animals

Healthy male mice, C57BL/6J species, weighing 25∼30 g, provided by the Beijing Vital River Lab Animal Technology Co. Ltd. (China), were used throughout this study. Animals were housed in environmentally controlled conditions (22°C, 12 h light-dark cycle with light cycle from 7∶00 am to 19∶00 pm and dark cycle from 19∶00 pm to 7∶00 am) with *ad libitum* access to standard laboratory mouse chow and water. The authors further attest that all efforts are made to minimize the number of animals used and their suffering.

### X-gal Staining

The complex of RDP and plasmid pVAX-Lac Z (RDP/pVAX-Lac Z ) in saline was intravenously injected into the mice (40 µg plasmid/mouse). The mice were respectively euthanized with overdose pentobarbital (45 mg/kg) at day 1, 3, 5 and 7 following intravenous injection. Three mice were used at the each indicated time point. The control mice received equal volumes (0.4 ml) of saline instead of plasmid. The liver, lung, kidney, spleen and brain of mouse were dissected out after saline perfusion to rinse out blood and the tissues were fixed in 4% paraformaldehyde in PBS (0.01 M, pH 7.4) for 48 h. Subsequently, the coronal sections (30 µm) were cut on a cryostat microtome. The method of X-gal staining was described previously [27], [28]. Briefly, the sections from different tissues were put in a 24-well plate and incubated with the freshly prepared X-gal solutions at 37°C until a detectable color was formed. The reaction was terminated by the addition of 1 mol/L Na_2_CO_3_. The sections were transferred to the slides, covered with coverslips and examined under a light microscope.

### Fluorescence Assays

N-terminal rhodamine B labeled RDP (Rho-RDP; purity >99%) was synthesized by Chinapeptides Co. Ltd. Each mouse received intravenously 40 µg of Rho-RDP/pVAX-Lac Z. Subsequently, the mice were euthanized at 2 h, 5 h and 12 h following injection. The brains were rapidly dissected and then frozen in liquid nitrogen. The cryostat sections were cut and the fluorescence images were obtained with emission wavelength of 550 nm (detection wavelength 610 nm) under a fluorescent microscope (*Olympus*, *Japan*).

### Western Blot Analysis

The complex of RDP and plasmid pVAX-BDNF (RDP/pVAX-BDNF) in saline was injected into mouse tail veins (40 µg plasmid/mouse). The mice were euthanized at day 1, 3, 5 and 7 after injection. The livers, kidneys and brains of mice were removed and frozen immediately in liquid nitrogen. The western blot was performed according to previous reports [29], [30]. In brief, the tissues of mice were homogenized in lysis buffer, and then the supernatant fluids were collected after centrifugation at 10 000 rpm for 20 min at 4°C. Samples were subjected to SDS-PAGE and electrophoretically transferred to PVDF membrane. The membrane was blocked in blocking solution for 2 h at room temperature, then immersed in goat anti-BDNF monoclonal antibody (Santa Cruz) overnight at 4°C. Membranes were washed twice for 15 min each in wash buffer and incubated for 2 h with horseradish peroxidase-conjugated secondary antibody, rabbit anti-goat IgG (1∶5000; Dingguo Biotechnology Co. Beijing, China). After washing twice for 15 min each in wash buffer, the signal was detected by the ECL system (Pierce). A western blot of GAPDH was performed in the same way, using a monoclonal GAPDH antibody (Kangcheng Biotechnology Co. Shanghai, China.) as the first antibody, and a goat anti-mouse horseradish peroxidase antibody as the second antibody.

### Immunofluorescence Staining of BDNF

The method of immunofluorescence staining was described previously [22]. In brief, mice were intravenously injected RDP, pVAX-BDNF or RDP/pVAX-BDNF (40 µg plasmid/mouse). At 3 day after injection, the mice were euthanized and brains were dissected out for cryostat sectioning. The slices were incubated in 10% normal goat serum diluted in PBS at 4°C overnight. The human BDNF monoclonal antibody was used as primary antibody, and FITC-labeled goat anti-human IgG as secondary antibody. The PBS was used to wash the slices before each addition. All immunostaining sections were observed with a fluorescence microscope (*Olympus* Optical *Co.*, Ltd., *Japan)*, and were photographed using the same magnification and identical color scale setting as a correction for background staining.

### Preparation of PD Model and Treatment

The mice were anesthetized with pentobarbital (30 mg/kg), and received a unilateral intra-cerebral injection of a total of 12 µg of 6-hydroxydopamine·HBr (Sigma, USA) dissolved in 0.02% ascorbic acid in 0.9% saline. The 6-hydroxydopamine (6 µg in 2 µL) was injected into the right striatum at 2 locations as described previously [31]. Mice were treated intravenously with saline, RDP, pVAX-BDNF or RDP/pVAX-BDNF (40 µg plasmid) at the day 2 after toxin injection, followed by once weekly for 2 weeks. The mice were euthanized at 3 weeks after toxin administration. Ten mice were used in each group.

### Behavioral Tests

Beginning 1 week after the toxin administration, mice were tested weekly for apomorphine- and amphetamine-induced rotation, which was performed on separate days. For the apomorphine testing, mice were injected apomorphine (0.6 mg/kg) subcutaneously. Full (360°), contralateral rotations only were counted over 20 min, starting 5 min after administration. For the amphetamine testing, mice were ip injected amphetamine (2.5 mg/kg). Full (360°), ipsilateral rotations only were counted over 20 min, starting 5 min after administration.

A vibrissae-elicited forelimb placing trial was carried out as described previously for mice [31], [32]. Each session included 120 trials (60 left side and 60 right side) in which a forelimb motor response to ipsilateral facial whisker stimulation was scored. In trials scored as a “3,” paw pads made full contact with table top. In trials scored as a “2,” paw pads do not make contact with the table. In trials scored as a “1” the limb moves forward only. In trials scored as a “0,” the limb does not move.

### Statistical Analysis

All data were given as mean ± SEM. For comparison between three groups a one-way analysis of variance (ANOVA) followed by the Tukey’s post hoc test was performed. A *p* value of <0.05 was considered to be statistically significant.

## Results

### RDP Binds and Delivers pVAX-Lac Z into the Brain

**Figure 1:**
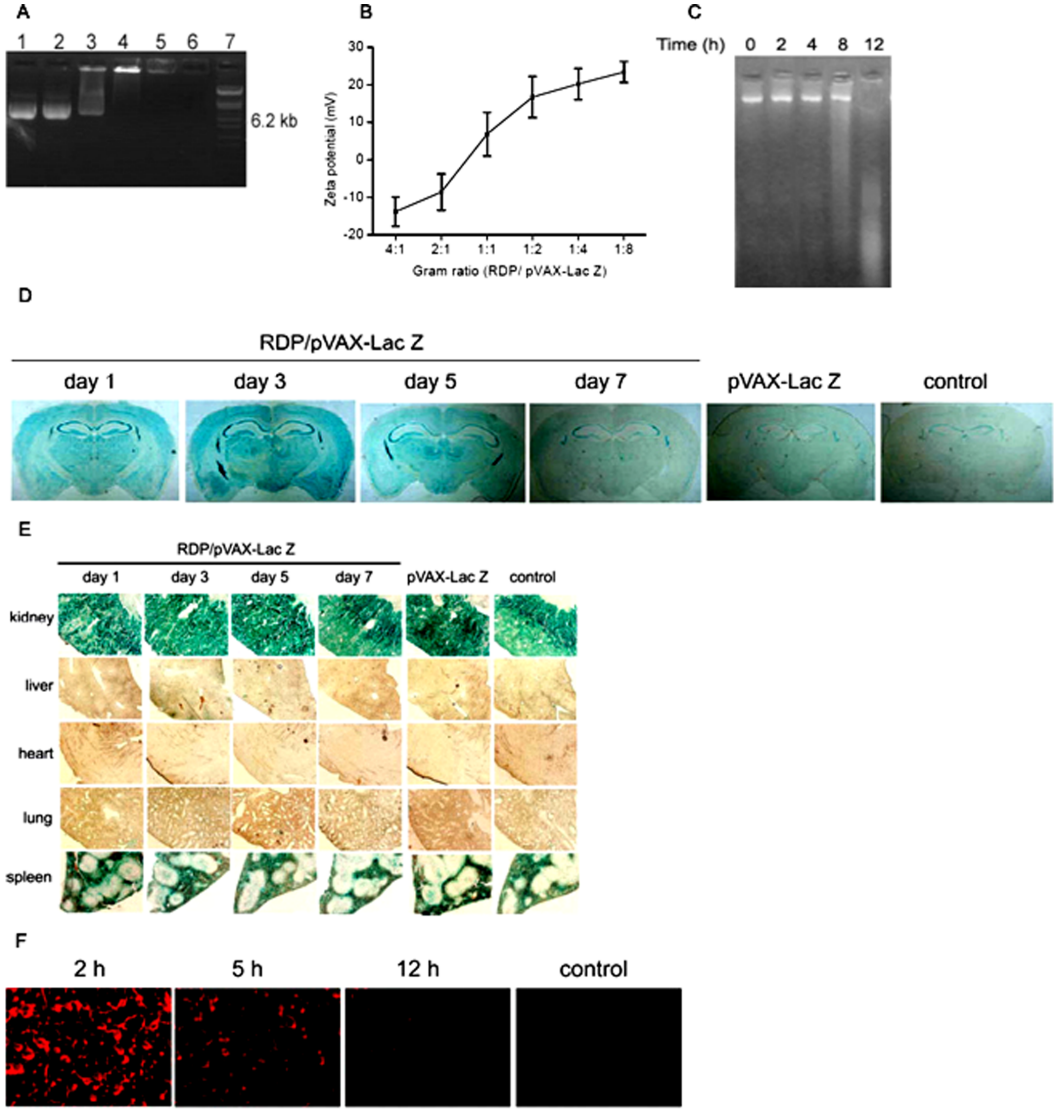
RDP binds and delivers plasmid pVAX-Lac Z into the brain. (*A*), Gel retardation assay. The plasmid pVAX-Lac Z (2 µg) was incubated with increasing amounts of RDP in saline. After 30 minutes at room temperature, aliquots were analyzed on a 1.0% agarose gel. The lanes 1 to 6 correspond to retardation assays in the presence of 0.5, 1, 2, 4, 8, 16 µg of the peptide; lane 7, DNA markers. (*B*), Zeta potential of RDP/pVAX-Lac Z complexes of different gram ratios. Data were expressed as mean ± SEM (n* = *3). (*C*), Serum stability of peptide and plasmid complex. The RDP/pVAX-Lac Z complexes were incubated with mouse serum at 37°C and analyzed by electrophoresis. Time points were analyzed at 0, 2, 4, 8 and 12 h. (*D*), Analysis of β-Gal gene expression assessed by X-gal staining in the brain. (*E*), X-gal staining in the peripheral tissues, including kidney, liver, heart, lung and spleen. Samples were analyzed at time points after intravenous administration. There were 3 mice per group. The results clearly showed that RDP mediated pVAX-Lac Z selectively into the brain and gene expression, while the plasmid without RDP did not cross the BBB when delivered in the same manner. (*F*), Fluorescence analysis of Rho-RDP as complexed with plasmid. Fluorescence was detected at mouse brain sections. Magnification 400.

We tested firstly whether RDP could bind and deliver plasmid into the brain. The result of gel retardation assay showed that RDP were able to bind plasmid in a dose-dependent manner (Fig. 1A), and a 1∶4 gram ratio of plasmid to peptide was found optimal for transduction. To characterize the peptide/DNA complexes, the charge was assessed using zeta potential. The results showed that the charge gradually increased with increasing amounts of cationic RDP peptides from −13.8±3.9 to 23.4±2.8 mV (Fig. 1B). Additionally, we examined whether RDP binding protected pVAX-Lac Z against degradation of serum nucleases. The result showed that RDP/pVAX-Lac Z complex was at least stable for up to 8 h in freshly prepared mouse serum. All of them suggested that the 1∶4 gram ratio of pVAX-Lac Z to RDP was optimal for transduction.

To determine transport efficiency and tissue specificity, the RDP/pVAX-Lac Z was intravenously injected into the mice, and X-Gal staining of tissues was used to test the gene expression and distribution of β-Gal. The results showed that exogenous β-Gal gene expression was detected only in the brain of RDP/pVAX-Lac Z treated mice and not in the detected peripheral organs, including kidney, liver, lung, heart and spleen (Fig. 1D and 1E), suggesting the specificity of brain targeting. In the brain, the staining was noted at day 1 and reached maximum at day 3 following RDP/pVAX-Lac Z injection, subsequently, the color became light at day 5, and almost disappeared at day 7 (Fig. 1D), suggesting that β-Gal gene expression lasted for about 1 week using RDP as plasmid delivery vector.

To analyze whether RDP entered neuronal cells as complexed with plasmid, Rho-RDP/pVAX-Lac Z was used and fluorescence of mouse brain sections was detected at different time points following complex administration. The results demonstrated that the fluorescence in cytoplasm was detected at 2 h, and gradually disappeared at 12 h (Fig. 1F), indicating that RDP/DNA complex could cross BBB and then enter the cells as a whole.

### RDP Delivers Plasmid Containing BDNF Gene into the Brain

**Figure 2:**
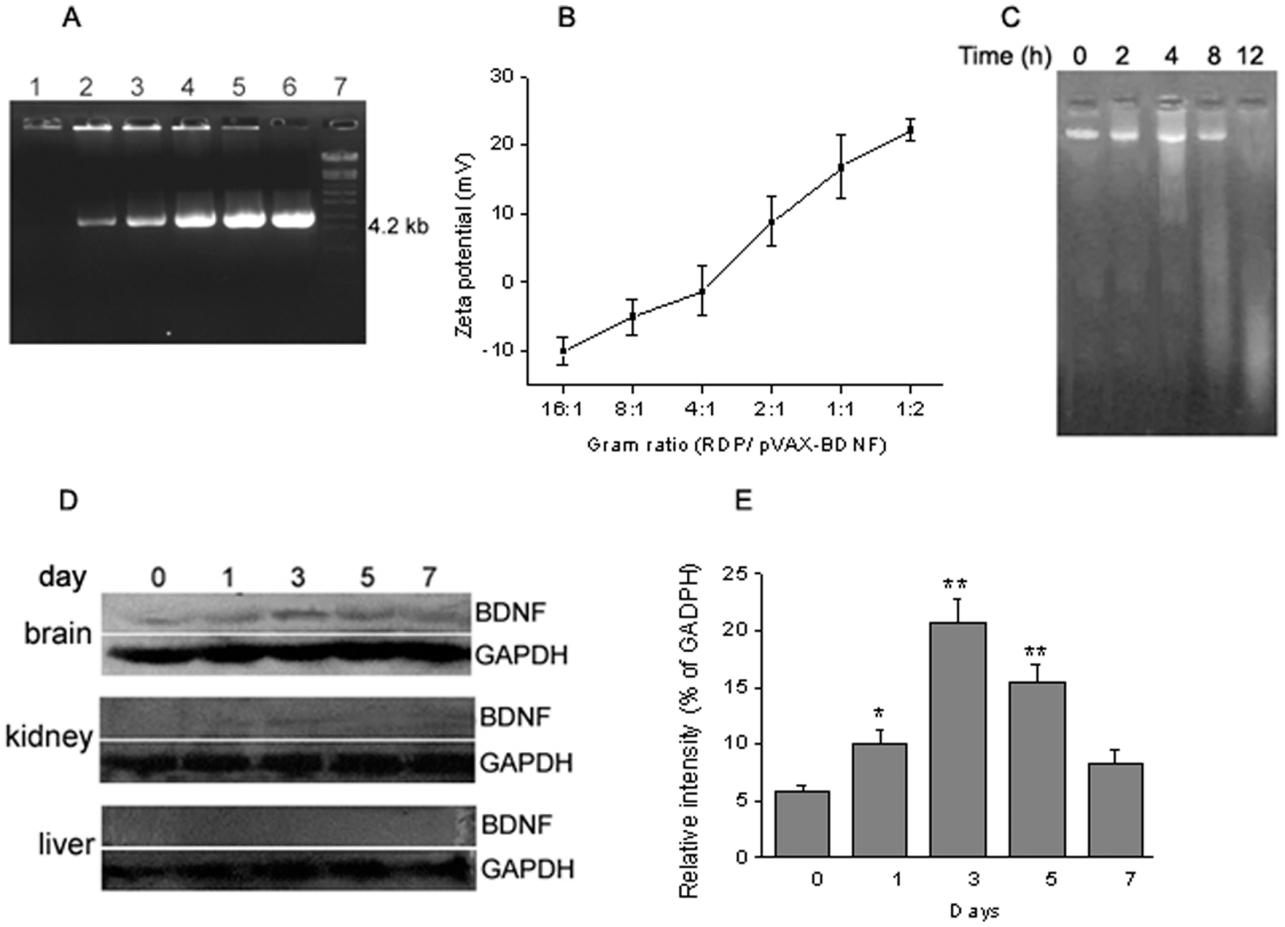
RDP delivers plasmid pVAX-BDNF into the brain and gene expression. (*A*), Gel retardation assay tests the migration pattern of plasmid DNA (2 µg) in the presence of RDP. The lanes 1 to 6 correspond to retardation assays of 4, 2, 1, 0.5, 0.25, 0.125 µg of RDP; lane 7, DNA markers. (*B*), Zeta potential of RDP/pVAX-EGFP complexes of different gram ratios (n* = *3). (*C*), Serum stability of peptide and plasmid complexes. Time points were analyzed at 0, 2, 4, 8 and 12 h following incubation with mouse serum at 37°C. (*D*), Western blot analysis of the BDNF in brain, kidney and liver of mice. The mice were intravenously injected with 40 µg complexes of DNA and plasmid, and the BDNF levels were detected on day 0. 1, 3, 5, 7 after injection. (E), Transient decrease of BDNF level in the brain. Data were expressed as mean ± SEM (n* = *3 in mice/time point). *p<0.05, **p<0.01 compared to the control (day 0),

To assay the optimal ratio for transduction, the plasmid (2 µg) containing BDNF gene was incubated with RDP at 16∶1, 8∶1, 4∶1, 2∶1, 1∶1 and 1∶2 gram ratios, respectively. The result suggested that the migration of pVAX-BDNF in agarose gel was completely retarded when the RDP/plasmid ratio of complex was 2∶1 (Fig. 2A). The result of zeta potential showed that the charge of RDP/pVAX-BDNF with 2∶1 gram ratio reached 22.2±1.6 mV (Fig. 2B). Also, the study of serum stability indicated that RDP/pVAX-BDNF complex of 2∶1 gram ratio was stable for at least 8 h in serum (Fig. 2C), in agreement with pVAX-Lac Z serum stability studies.

**Figure 3:**
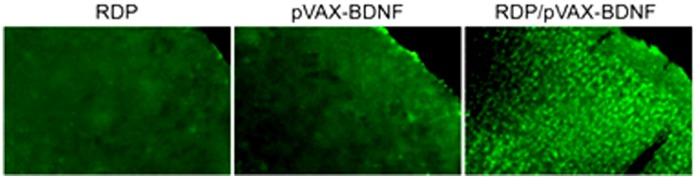
Representative photographs of BDNF immunostraining in the brain. Magnification 100. The mice were respectively injected RDP, pVAX-BDNF and RDP/pVAX-BDNF (three mice in per group) and were euthanized at 3 day after injection.

To confirm the ability of brain-targeting DNA delivery of RDP, we also test whether RDP can deliver a potential therapeutic plasmid encoding BDNF gene into the brain. Western blot and immunofluorescence staining were used to examine BDNF gene expression after the complex of RDP/pVAX-BDNF injection. The results of western blot analysis showed that the BDNF levels were significantly increased in the brains at day 3 and 5 after administration (Fig. 2D), whereas no signal was detected in mouse liver and kidney, the two main organs responsible for drug distribution and metabolism. Additionally, the result of immunofluorescence staining confirmed the brain delivery of plasmid complexed to RDP. A number of immunopositive cells were detected in the brains of RDP/pVAX-BDNF treated mice, and the staining appeared in the whole BDNF-positive cell body, whereas there were few fluorescence positive cells in the control groups (Fig. 3). These results indicate that RDP enables the intravenous delivery of plasmid to gene expression within the brain.

### Binding of Plasmids Affects the Conformation Structure of RDP

**Figure 4:**
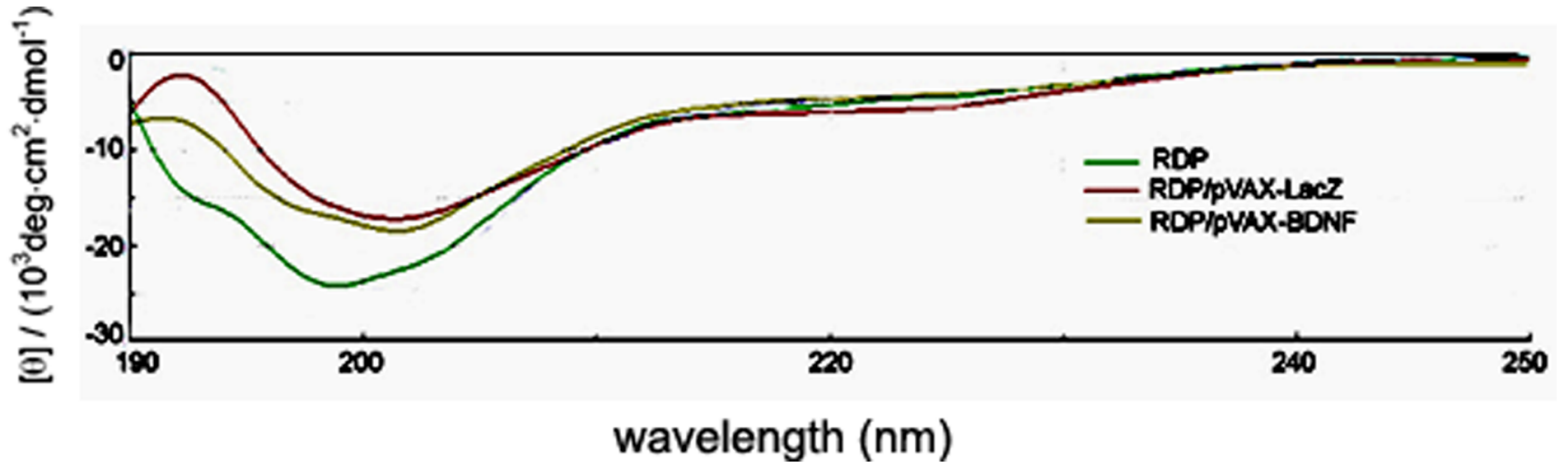
CD spectra of RDP with or without the plasmid DNA. The peptide (0.2 mg/ml) was dissolved in distilled water. Measurement was performed in the presence or absence of the plasmid.

To analyze the structural features of RDP that bound to the plasmid DNA, CD spectra of the peptide were measured. The results suggested that there were significant differences between the secondary structures of free RDP and DNA-binding peptides (Fig. 4). The CD spectrum taken for free RDP showed the propensity to form random coil (51.9%) and β-sheet (35.7%) in aqueous solution, and an increase in the amount of β-sheet contents after binding the plasmid pVAX-Lac Z (54.4%) and pVAX-BDNF (51.8%).

### The Therapeutic Effect of RDP/pVAX-BDNF in Experimental PD Mice

**Figure 5:**
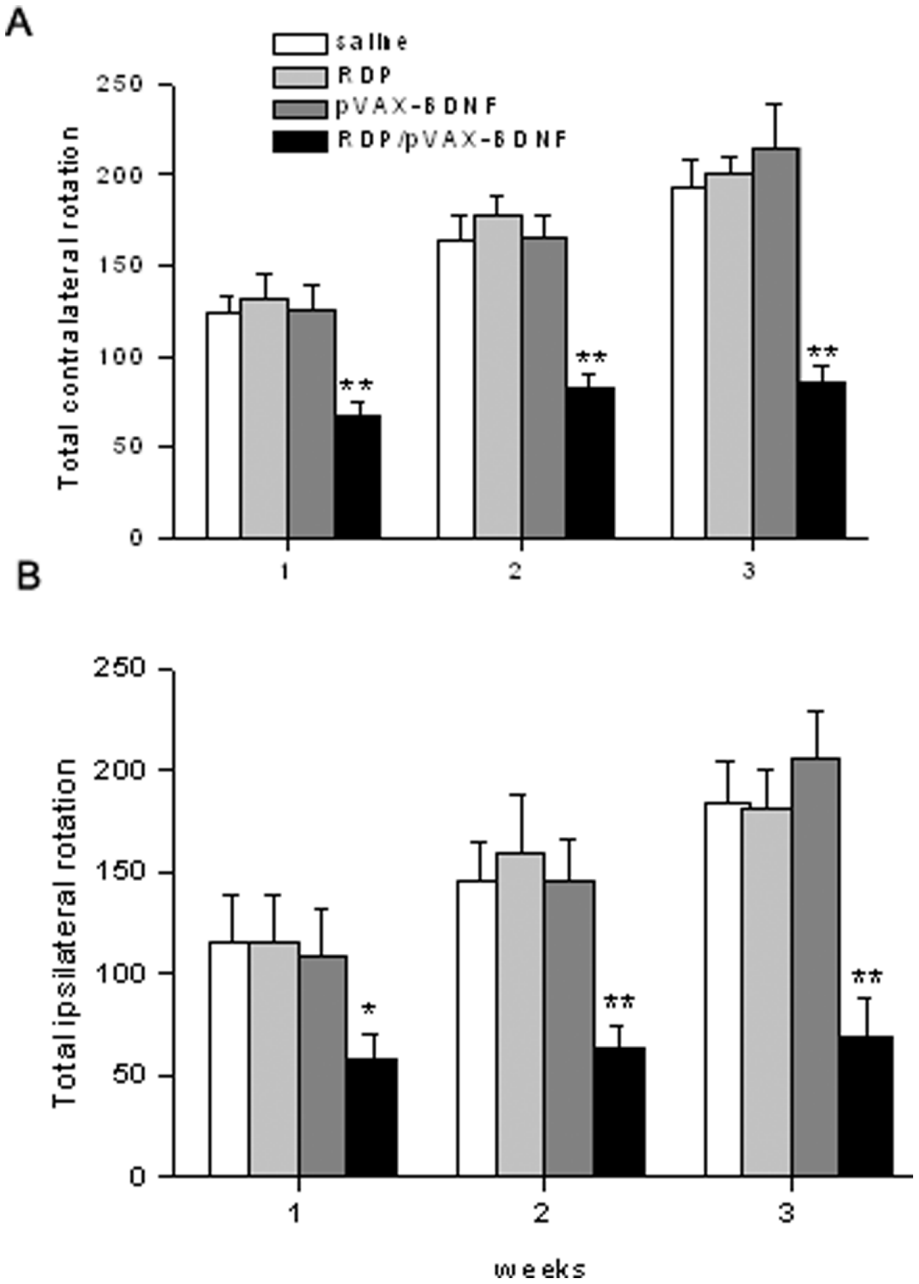
Therapeutic effect of RDP/pVAX-BDNF on experimental PD. Rotation measurements following the administration of either apomorphine (*A*) or amphetamine (*B*) for PD mice treated with saline, RDP, pVAX-BDNF or RDP/pVAX-BDNF. Treatment was administered at once weekly for 3 weeks after toxin injection. Data are mean ± SEM (n = 10 mice/group). *p<0.05, **p<0.01 compared to the saline treated animals.

Experimental PD model was induced in mice by the intra-striatal injection of 6-hydroxydopamine. Mice were tested for apomorphine- and amphetamine- induced rotation at 1, 2, and 3 weeks after toxin administration. The results were shown in Fig. 5. The mice treated with saline, RDP or pVAX-BDNF exhibited an increase in apomorphine- and amphetamine-induced rotation at 1–3 weeks after toxin injection. However, the mice treated with the RDP/pVAX-BDNF showed significant decreases in both apomorphine- and amphetamine-induced rotation compared to the saline group, suggesting that the mice in the RDP/pVAX-BDNF group had a marked improvement in induced rotation behaviors.

**Figure 6:**
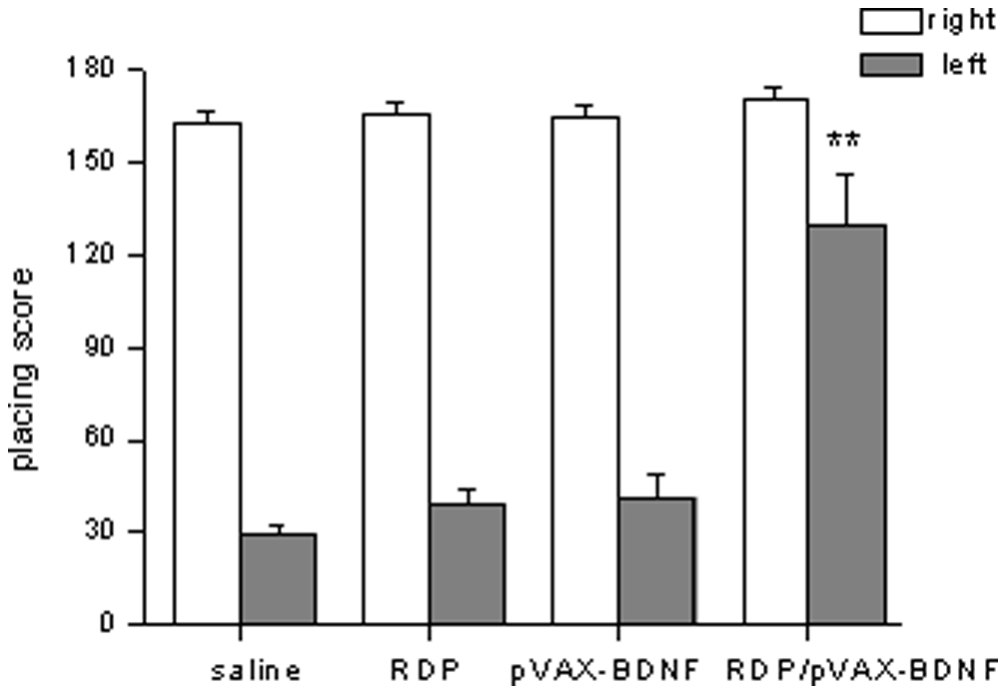
Vibrissae-elicited forelimb placing test. Neurologic deficit is partly recovered in RDP/pVAX-BDNF treated mice. Data are mean ± SEM (n = 10 mice/group). *p<0.05, **p<0.01 compared to the saline treated animals.

At the end of the study, the mice were evaluated with the vibrissae-elicited forelimb placing test to identity the pharmacologic efficacy of the RDP/pVAX-BDNF gene therapy. The unilateral 6-hydroxydopamine lesioned mouse failed to place the forelimb contralateral to the lesion. The results showed that all of mice in the different groups had maximal placing scores on the right side, which is ipsilateral to the side of toxin injection (Fig. 6). The mice treated with saline, RDP or pVAX-BDNF showed a reduction in placing score on the lesioned side, while the mice treated with RDP/pVAX-BDNF showed a significant increase in placing score, relative to the saline treated mice, on the lesioned side (Fig. 6).

## Discussion

The development of peptide delivery systems that are capable of efficient delivery macromolecules across BBB is essential for the clinical application of gene and protein therapy to brain diseases. The present study demonstrates that RDP as a single-component plasmid transfer vector can efficiently deliver DNA into the mouse brain by systemic administration.

The use of cationic peptides for gene delivery is particularly interesting because they are able to efficiently condense DNA due to electrostatic interaction [33], [34]. RDP is a water soluble cationic peptide with pI of 12.3, including 33.3% basic amino acids (12 Arg and 1 Lys). The negatively charged plasmid DNA and the positively charged RDP can bind through electrostatic interactions in saline and form complexes. The gel retardation assays revealed that 1 µg plasmid pVAX-Lac Z was retained in the presence of 4 µg of RDP, and 2 µg of peptide provided approximately the number of positively charged μ-amino groups necessary to neutralize the negative charges of the phosphate moieties in the engaged amount of plasmid pVAX-BDNF. The CD spectra also revealed the distinct conformational transition from random coil to β-sheet when RDP bound to plasmid DNA, indicating that electrostatic interaction forces between peptide and DNA might be responsible for this conformational transition.

The formation of peptide/DNA complexes was confirmed by zeta potential measurements, and the results indicated that the zeta potential of peptide/DNA complexes increased gradually with increasing amounts of RDP peptide in a dose-dependent manner, and reached about +20 − 25 mV at 4∶1 gram ratio of RDP to pVAX-Lac Z or at 2∶1 gram ratio of RDP to pVAX-BDNF.

The stability of a DNA formulation in serum is fundamental to its successful application in vivo, since premature metabolism results in the generation of fragmented DNA which lacks gene transfer potency [35]. An examination of the serum stability of peptide/DNA complexes determined that the DNA was at least stable for up to 8 h in mouse serum, demonstrating the protection of plasmid by RDP against the degradation of serum nucleases. Thus, peptide/DNA complexes could be used for transfection in vivo at certain ratio of RDP to plasmid.

For potential delivery in vivo, the gene transfection efficiency and long-term expression of reporter gene Lac Z were evaluated in the mice after iv administration of RDP/pVAX-Lac Z complex. The results showed a high level of β-gal expression in brain at a plasmid dose of 40 µg. In addition to the high gene expression in vivo, Lac Z had a potential to prolong gene expression up to 7 days. The results suggest that RDP could be used as a powerful tool for the delivery of plasmid DNA.

BDNF is a member of the neurotrophin family, and involved in nerve growth and survival. BDNF also enhance the survival of dopaminergic neuron, and induces striatal neurogenesis in adult rats with 6-hydroxydopamine lesions [25]. In the animal behavior tests, BDNF gene therapy can promote functional recovery of rats from 6-hydroxydopamine lesions as mediated by an adeno-associated virus vector [36]. In this study, we used RDP as a non-viral gene vector to deliver plasmid containing BDNF gene to the brain, and the genes were efficiently expressed in the neuronal cells. Also, BDNF gene expression could produce neuroprotective effect in mice with 6-hydroxydopamine lesions, as shown by elevated rotational behaviors and whisker-induced forelimb placement test.

The internalization mechanism of RDP is not very clear. It’s known that rabies virus interacts specifically with the nicotinic acetylcholine receptor (nAchR) on neuronal cells to enable viral entry into neuronal cells, and RVG29-d9R deliver siRNA through the α7 subtype of nAchR mediated transcytosis [20], [21]. Additionally, previous studies reported that water soluble peptides with random coil and β-sheet conformation could be internalized into cells in an energy-, temperature- and pH-dependent manner [37], [38]. Here we showed that Rho-RDP/DNA could penetrate BBB and then enter neuronal cells as a whole, and RDP was located in cytoplasm at least 5 h following administration, implying that transcytosis might mediate the cellular internalization of RDP/DNA. We have further identified that specific GABA receptors on neuronal cells, instead of nAchR, mediates the RDP and its complexes transfer into the cells through energy-depending endocytosis (data not shown). GABA is a common neurotransmitter whose receptors are expressed in all major brain structures and brain capillary endothelial cells [39]. The specific cellular uptake of RDP/DNA complex can be inhibited by free RDP and GABA but not by AchR agonists/antagonists, indicating that the transport of RDP probably relates to the GABA receptor.

Plasmids are highly desirable DNA vectors for gene therapy, as they offer multiple advantages over viral vectors, including large packaging capacity, stability without integration and reduced toxicity [40], [41]. However, plasmid is extremely difficult to be transvascularly delivered into the brain using of known techniques [42]. Here we used a simple and effective approach to achieve exogenous DNA into the brain through systemic administration. The DNA delivery mediated by RDP not only crosses the mouse BBB in vivo, but objective gene is specifically expressed in the brain.

The present report also suggests that intravenous injection of RDP/pVAX-BDNF can significantly improve rotation behaviors in toxin-induced PD model. Moreover, no toxic reaction was observed in mice during experiment period after RDP systemic administration, and plasmid pVAX is the only vector authorized by FDA for gene therapy in clinical trial, so this approach might be a potential new gene treatment for human brain diseases.
